# The COP9 Signalosome Converts Temporal Hormone Signaling to Spatial Restriction on Neural Competence

**DOI:** 10.1371/journal.pgen.1004760

**Published:** 2014-11-13

**Authors:** Yi-Chun Huang, Yu-Nung Lu, June-Tai Wu, Cheng-Ting Chien, Haiwei Pi

**Affiliations:** 1 Graduate Institute of Life Sciences, National Defense Medical Center, Taipei, Taiwan; 2 Department of Biomedical Sciences, College of Medicine, Chang Gung University, Tao-Yuan, Taiwan; 3 Insitute of Molecular Biology, Academia Sinica, Taipei, Taiwan; 4 Institute of Molecular Biology, National Taiwan University, Taipei, Taiwan; Baylor College of Medicine, United States of America

## Abstract

During development, neural competence is conferred and maintained by integrating spatial and temporal regulations. The *Drosophila* sensory bristles that detect mechanical and chemical stimulations are arranged in stereotypical positions. The anterior wing margin (AWM) is arrayed with neuron-innervated sensory bristles, while posterior wing margin (PWM) bristles are non-innervated. We found that the COP9 signalosome (CSN) suppresses the neural competence of non-innervated bristles at the PWM. In *CSN* mutants, PWM bristles are transformed into neuron-innervated, which is attributed to sustained expression of the neural-determining factor Senseless (Sens). The CSN suppresses Sens through repression of the ecdysone signaling target gene *broad* (*br*) that encodes the BR-Z1 transcription factor to activate *sens* expression. Strikingly, CSN suppression of BR-Z1 is initiated at the prepupa-to-pupa transition, leading to Sens downregulation, and termination of the neural competence of PWM bristles. The role of ecdysone signaling to repress *br* after the prepupa-to-pupa transition is distinct from its conventional role in activation, and requires CSN deneddylating activity and multiple cullins, the major substrates of deneddylation. Several CSN subunits physically associate with ecdysone receptors to represses *br* at the transcriptional level. We propose a model in which nuclear hormone receptors cooperate with the deneddylation machinery to temporally shutdown downstream target gene expression, conferring a spatial restriction on neural competence at the PWM.

## Author Summary

A critical step in building a functional nervous system is to generate neurons at the appropriate locations. Neural competence is acquired at the precursor stage with the expression of specific transcription factors. One such critical factor is Senseless (Sens), as precursors lacking Sens fail to develop to neurons. Here we describe the critical role of protein complex COP9 signalosome (CSN) that regulates Sens expression by integrating temporal and spatial information. This was studied in developing *Drosophila* wing tissues, in which the anterior wing margin develops neuron-innervated bristles, while the posterior wing margin develops non-innervated bristles. The CSN complex is required for the anterior-posterior difference in spatial patterning of neuron formation, and posterior cells lacking CSN develop innervated bristles like anterior cells. CSN accomplishes this by transforming the temporal hormonal ecdysone signaling from activation to repression of downstream target BR-Z1. As BR-Z1 itself is a transcription activator, repression of BR-Z1 in turn leads to repression of Sens in posterior wing margin, eventually terminating the neural competence. Repression of BR-Z1 expression requires the interaction between the CSN complex and the ecdysone receptors. Our results suggest a novel CSN-mediated regulation that converts temporal hormone signaling to the patterning of neurons at the right place.

## Introduction

Neural specification generates diverse neural cells that are located at exact positions necessary for specialized functions. It is well established that positional cues, such as extrinsic signals and intrinsic tissue-specific transcriptional factors, play key roles in neural specification and neuronal patterning. In *Drosophila*, the sensory bristles occupy stereotypical positions on the head, notum, abdomen, legs and anterior wing margins (AWMs). Patterning of sensory bristles is primarily defined by the spatially restricted expressions of bHLH proneural proteins Achaete (Ac) and Scute (Sc). High-level Ac and Sc expressions in the presumptive sensory organ precursors (SOPs) activate genetic programs for further specification and differentiation, leading to the generation of sensory bristles comprising neuron, sheath, hair, and socket cells [1], [2]. A key target of the proneural proteins, the Zinc finger protein Senseless (Sens), is turned on in smaller subsets of proneural cells, and the expression levels are further elevated in SOPs and SOP lineage cells [3]. Sens is required for the specifications of SOPs and SOP daughter cells pIIa [3], [4]. Misexpression of Sens in epithelial cells is sufficient to induce sensory organ formation in a process bypassing the requirement of proneural proteins, indicating that Sens plays a key role in sensory organ formation [4].

In addition to spatially restricted expression of transcription factors, specification of SOPs also depends on steroid hormonal ecdysone (20-hydroxyecdysone, 20E) signaling. For example, ecdysone signaling is required for the specification of chemosensory bristles at the AWM at late third instar larval stages [5]. Pulses of ecdysones set temporal boundaries in developmental processes like inducing molting and metamorphosis [6]–[10]. Ecdysone signaling is mediated through the heterodimeric complex between the ecdysone receptor EcR and the RXR ortholog Ultraspiracle (USP) that binds to the ecdysone response element (EcRE) [11], [12]. Ligand binding transforms the EcR/USP complex from a repressed to active state, leading to transcriptional activation of a hierarchy of target genes. Among them, *broad* (*br*) is the key early gene that mediates ecdysone signaling in the induction of metamorphosis. The *br* gene encodes four isoforms BR-Z1 to BR-Z4 through alternative splicing [13], [14]. In *br* mutants, responses of a large set of early and late genes to ecdysone signaling are abrogated [15], [16], and mutants die as wandering larvae unable to initiate puparium formation [17], [18]. Dysregulation of *br* also disrupts sensory neuron differentiation and suppresses adult abdominal cuticle formation [5], [19]. Therefore, tight regulation of *br* expression is crucial for development of various insect tissues.

The COP9 signalosome (CSN) is a highly conserved protein complex initially identified in *Arabidopsis* for suppression of photomorphogenesis [20]–[22]. Subsequent identification and characterization in mammalian cells, insects and yeast reveal that the CSN complex participates in diverse cellular and developmental processes (for reviews, see references [23] and [24]). The major CSN function is to deconjugate the ubiquitin-like peptide Nedd8 from cullins, the scaffolding proteins in cullin-RING ubiquitin ligases (CRLs) [25]. CSN-mediated deneddylation of cullins inactivates ubiquitin ligase activity and protects CRLs from turnover. Thus, cycling between neddylation and deneddylation maintains the physiological CRL activity [26], [27]. Additional CSN-associated activities such as deubiquitination and phosphorylation are linked to the maintenance of target protein stability [28]–[30].

Studies in *Drosophila* have revealed several CSN functions in development and adult physiology. Transcriptome analysis has shown a role for CSN4 and CSN5, two subunits of the CSN complex, in transcriptional repression of developmentally regulated genes [31]. *CSN4* mutants show defect in larval molting while *CSN5* mutations induce melanotic formation at larvae stages [32]. The CSN complex regulates protein degradation of CycE and Timeless during oogenesis and circadian rhythm, respectively [33], [34]. By regulating Cul1 and Cul3, the CSN complex has dual functions in dendrite morphogenesis [35], [36]. Interestingly, the CSN activity confers specifically the intermediate response in graded Hedgehog signaling [37]. In an RNAi-based screening to identify genes involved in Notch signaling, CSN subunits were identified to be essential in binary cell fate determination in sensory organ development [38].

The bristles along the anterior and posterior wing margin succumb to different fates; AWM bristles are innervated by neurons beneath the cuticle while posterior wing margin (PWM) bristles are non-innervated. Both types of bristles require transcription factors Sens and the bHLH protein Daughterless for precursor cell selection during third instar larval and prepupal stages [4]. However, it is still unclear how the neural competence of PWM bristles is suppressed to prevent formation of neurons and associated neural cells. We have identified a role of the CSN complex in inhibition of the neural competence of non-innervated bristles through Sens downregulation at the onset of pupal development. Activation of ecdysone signaling at the prepupa-to-pupa transition is required for pupal BR-Z1 suppression, which leads to Sens suppression. The CSN complex and ecdysone receptors act in the same genetic pathway and form protein complexes to block BR-Z1 expression in pupal wings. Thus, our results demonstrated that the CSN complex acts as a nexus to convert temporal hormone stimulation to spatial restriction of neural competence.

## Results

### Transformation of PWM bristles into innervated sensory bristles in *CSN* mutants

**Figure 1:**
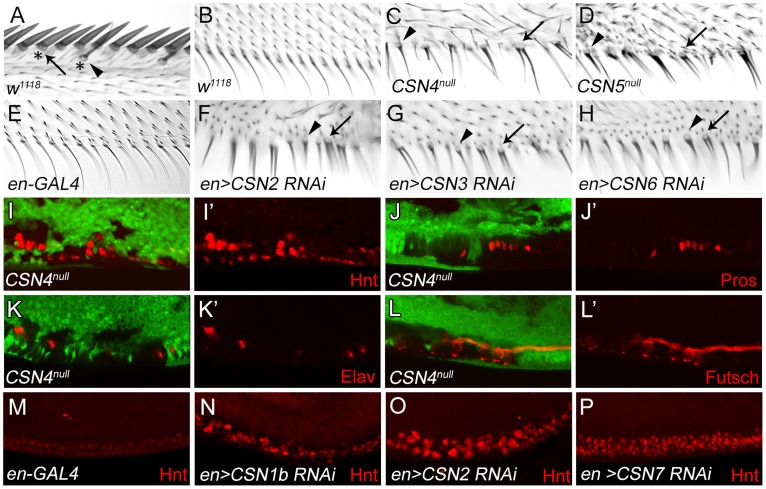
Transformation from non-innervated to innervated PWM bristles in *CSN* mutants. (A) AWM innervated bristles. Arrow and arrowhead indicate stout mechanosensory and chemosensory bristles, respectively. Also indicated are dome-shape sockets (asterisks) surrounding shafts. (B) PWM non-innervated bristles. (C–H) PWM bristles in *CSN4^null^* (C), *CSN5^null^* (D), *en-GAL4* control (E), *CSN2* RNAi (F), *CSN3* RNAi (G) and *CSN6* RNAi (H) driven by *en-GAL4* driver. Arrowheads indicate single bristles with a thicker shaft and a socket. Arrows indicate single bristles with two shafts and one large socket. (I–L′) *CSN4^null^* clones, located at the PWM and marked by the absence of GFP, exhibited elevated Hnt expression 20–24 h APF (red in I, I′), and ectopic expression of Pros 24–28 h APF (red in J, J′), Elav 28–32 h APF (red in K, K′) and Futsch 28–32 h APF (red in L, L′). (I′, J′, K′, L′) Single-channel images. (M–P) Hnt (red) expressions 20–24 h APF at the PWM of *en-GAL4* control (M), and *CSN1b* RNAi (N), *CSN2* RNAi (O) and *CSN7* RNAi (P) by *en-GAL4*.

Innervated sensory bristles, including stout and slender mechanosensory and spaced chemosensory bristles, are located along the AWM of wild-type wings. These sensory bristles grow dome-shaped sockets at the base (Figure 1A), and are innervated by neurons [39]. Non-innervated bristles along the PWM, however, display only thin hairs without sockets (Figure 1B). In *CSN4^null^* and *CSN5^null^* mutant clones, PWM bristles adopted morphological characteristics of innervated sensory bristles. Mutant bristles had thicker hairs surrounded by sockets (arrowheads in Figure 1C, 1D). Some bristles had double hairs emerging from one large socket (arrows), reminiscent of the double hair/double socket phenotype caused by fate transformation of SOP lineage cells [40], [41]. Similar morphological changes of PWM bristles were also observed in wings expressing interference RNA (RNAi) by *en-GAL4* to knockdown *CSN1b*, *CSN2*, *CSN3*, *CSN6* and *CSN7* in the posterior compartment (Figure 1F–H, and Figure S1A, S1B), suggesting that these CSN subunits function together as a holoenzyme to suppress formation of innervated bristles at the PWM.

The altered developmental process of PWM bristles in *CSN* mutants was examined for the expression of nuclear protein Hindsight (Hnt)/Pebbled in bristle lineage cells [42]. In wild-type wing discs at 20–24 hours (h) after puparium formation (APF), clusters of AWM innervated bristle lineage cells had larger nuclei and expressed high levels of Hnt, while PWM non-innervated bristle cells, with smaller nuclei, expressed low levels of Hnt (Figure S1C–C″). In *CSN4^null^* and *CSN5^null^* PWM clones, cells with larger nuclei and high-level Hnt were observed (Figure 1I, 1I′ and Figure S1D, S1D′). Accumulations of Hnt in PWM cells were also detected in *CSN1b*, *CSN2* and *CSN7* knockdowns by *en-GAL4* (Figure 1M–P). While non-innervated PWM bristles contain no neurons or sheath cells [4], [39], these two types of cells were detected in PWM clones for *CSN4^null^*, as shown by Prospero (Pros) expression in pIIb precursors and sheath cells [43] and Elav expression in neurons [44] (Figure 1J–K). These ectopic neurons extended axons as revealed by immunostaining for the microtubule-associated protein Futsch (Figure 1L, 1L′). Taken together, these morphological and molecular analyses indicate that the CSN inhibits the neural potential of PWM bristles.

### The CSN suppresses Sens independent of proneural genes *ac* and *sc*

**Figure 2:**
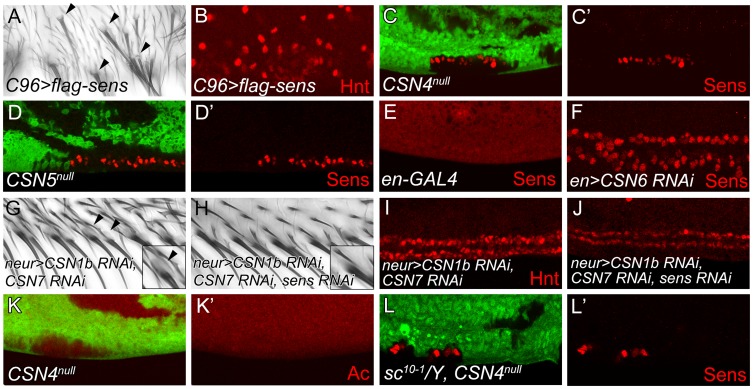
The CSN suppresses Sens to inhibit neural differentiation of PWM bristles. (A, B) Overexpression of *sens* in *C96-GAL4*-driven *UAS-flag-sens* resulted in ectopic innervated bristles at the PWM of adult wings (A) and ectopic Hnt-positive cells at the PWM of wing discs 20–24 h APF (B). (C–F) Sens (red) expression was detected in *CSN4^null^* clones (C, C′), *CSN5^null^* clones (D, D′) and *CSN6* RNAi knockdown by *en-GAL4* (E, F) at the PWM 20–24 h APF. (G, H) Innervated bristles with sockets (arrowheads) appeared at the PWM of *neur-GAL4* double knockdown of *CSN1b* and *CSN7* (G), which were no longer detected when *sens* was also knocked down (H). (Insets in G, H) Enlarged figures showing single bristle and socket (arrowhead). (I, J) Double knockdown of *CSN1b* and *CSN7* by *neur-GAL4* resulted in accumulation of higher-level Hnt at the PWM, which was suppressed by *sens* knockdown (J). (K, K′) Ac (red) was not expressed in *CSN4^null^* clones in wing disc 18–22 h APF. (L, L′) Ectopic Sens-positive cells were detected in PWM clones for *CSN4^null^* in *sc^10-1^* null mutant disc 20–24 h APF.

Overexpression of Sens in epithelial cells can induce innervated bristles [3]. As shown, overexpression of Sens by *C96-GAL4* along the wing margin induced bristles with sockets around the PWM (Figure 2A, arrowheads). Concomitantly, high levels of Hnt expressions were also detected (Figure 2B), confirming that ectopic Sens expression induces innervated bristles at the PWM.

With the induction of innervated sensory bristles by high levels of Sens, we then examined whether Sens was upregulated in *CSN* mutants. In *CSN4^null^* and *CSN5^null^* PWM cells, the Sens levels were elevated (Figure 2C–D′). The elevation of Sens expression was also detected in *CSN6*, *CSN2* and *CSN3* knockdown cells, suggesting that CSN functions as a complex to suppress Sens expression (Figure 2E, 2F and Figure S2A, S2B). Next we addressed whether Sens is required for innervated bristle formation in *CSN* mutant cells at the PWM. Double knockdown of *CSN1b* and *CSN7* in bristle lineage cells by *neur-GAL4* resulted in socket formation in PWM bristles (Figure 2G), ectopic Sens expression (Figure S2C), and consistently, higher levels of Hnt in PWM cells with large nuclei (Figure 2I). When *sens* was knocked down simultaneously (Figure S2D), those features of innervated bristles in *CSN* mutants were suppressed. Socket morphology was no longer visible (Figure 2H), and most PWM cells had smaller nuclei and expressed lower levels of Hnt (Figure 2J). Thus, Sens induction in *CSN* mutant cells is required for the formation of innervated bristles at the PWM.

The proneural proteins Ac and Sc are specifically expressed in proneural clusters and precursors of AWM chemosensory bristles [45]. Formation of innervated sensory bristles in *CSN* mutant cells could be the consequence of ectopic proneural protein induction. However, Ac expression was not induced in *CSN4^null^* and *CSN5^null^* PWM cells. (Figure 2K, 2K′ and Figure S2E–F′). Also, removal of *ac* and *sc* in *CSN4^null^* clones still accumulated Sens in PWM cells (Figure 2L, 2L′). Thus, proneural proteins Ac and Sc are not required for Sens upregulation in *CSN* mutant cells at the PWM.

### Sens accumulation is induced by BR-Z1 upregulation in *CSN* mutant cells

**Figure 3:**
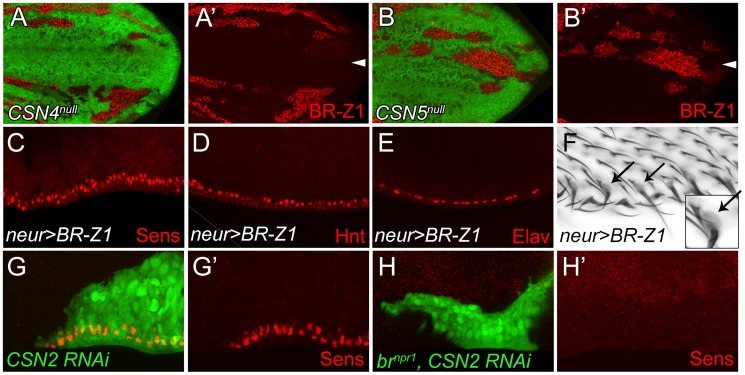
BR-Z1 upregulation is required for Sens upregulation at the PWM of *CSN* mutants. (A–B′) Upregulation of BR-Z1 (red) in *CSN4^null^* (A, A′) and *CSN5^null^* clones (B, B′) 20–24 h APF. White arrowheads indicate the anterior-posterior boundary. (C–F) Transient overexpression of BR-Z1 in bristle lineage cells via *neur-GAL4* and *Tub-GAL80^ts^*. (C–E) Ectopic expression of Sens (red in C), accumulation of Hnt (red in D) and ectopic formation of Elav-positive neurons (red in E) were observed at the PWM 22–24 h APF. Prepupae grown at 18°C were shifted to 37°C for one hour at 8–10 h APF, and then incubated at 29°C until dissection. (F) Bristles with dome-shape sockets (indicated by arrows) were observed at the PWM. Inset: enlarged figure showing single bristle. The experiment was carried out similarly to (C–E) except for incubation at 29°C for 12 hours, and returning back to 18°C until eclosion. (G–H′) Knockdown of *CSN2*, in MARCM clones (green), induced Sens (red) upregulation at the PWM 20–24 h APF (G, G′), which was abolished in *CSN2* RNAi *br^npr1^* double mutant MARCM clones (green) (H, H′).

The Zinc-finger transcriptional factor BR-Z1 induces Sens expression in response to ecdysone signaling in chemosensory precursors [5]. Thus, we tested if the CSN also downregulates BR-Z1, leading to the suppression of Sens in PWM cells. As expected, the BR-Z1 levels were strongly elevated in *CSN4^null^* and *CSN5^null^* wing-disc cells 20–24 h APF (Figure 3A–B′). Elevated BR-Z1 levels were also detected in *CSN2*-knockdown cells (Figure S3A). Distinct from Sens regulation, CSN suppression of BR-Z1 was ubiquitous in all wing-disc cells, not restricted to the wing margin. Furthermore, forced expression of BR-Z1 in bristle lineage cells by *neur-GAL4* elevated Sens and Hnt expression and induced Elav-positive neurons at the PWM (Figure 3C–E). In adult wings, all bristles at the PWM had dome-shape sockets (Figure 3F), suggesting that BR-Z1 promotes the formation of innervated bristles at the PWM. The *br* locus encodes three additional isoforms BR-Z2, BR-Z3 and BR-Z4, and functional redundancy was observed among these isoforms [46], [47]. We found that the activity to induce innervated bristles is not limited to BR-Z1, as overexpression of BR-Z3 also promoted Sens accumulation and innervated bristle formation at the PWM (Figure S3B, S3C).

Lastly, we examined the role *br* plays in promotion of innervated bristle formation when *CSN* activity is abrogated. To completely inactivate all *br* isoforms to prevent functional redundancy, the *br* null allele, *br^npr1^*, was used. In control clones, Sens expression was elevated in *CSN2 RNAi* cells (Figure 3G, 3G′). However, Sens elevation was no longer detected in *CSN2 RNAi* cells when *br* was simultaneously inactivated (Figure 3H, 3H′). These results unequivocally indicate that *br* is the critical factor required in *CSN* mutant cells to upregulate Sens expression.

### CSN regulation of BR-Z1 and Sens levels is time-dependent

**Figure 4:**
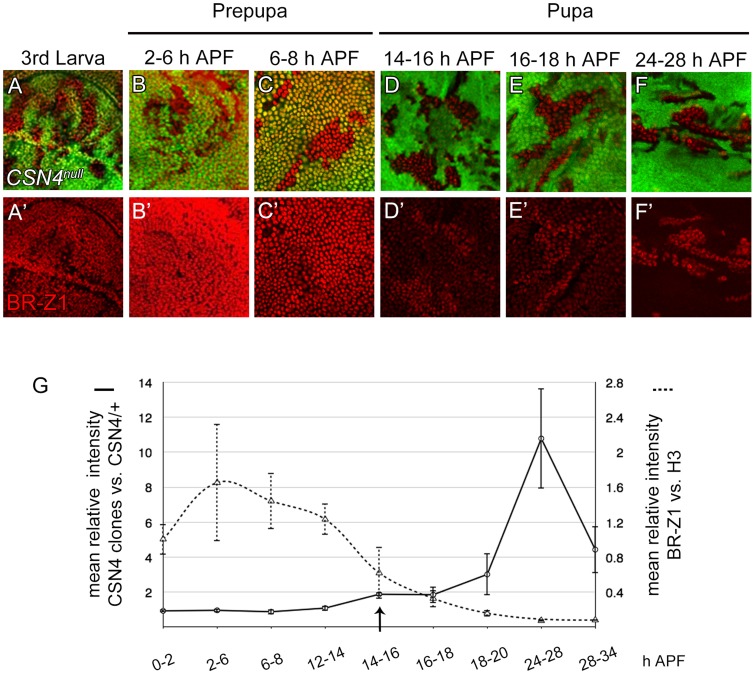
The CSN suppresses BR-Z1 expression after prepupa-to-pupa transition. (A–F′) BR-Z1 (red) expression in *CSN4^null^* clones in wing discs from late third-instar larval to pupal stages 24–28 h APF. (A–C′) BR-Z1 expression was not affected in *CSN4^null^* clones at late third larval instar (A, A′) and prepupal stages (B–C′). (D–F′) BR-Z1 levels in *CSN4^null^* clones were upregulated at pupal stages. (G) Diagram showing the mean anti-BR-Z1 immunofluorescent intensity from 0 to 34 h APF. The dashed line represents the mean relative intensities of anti-BR-Z1/anti-H3 in wild-type *w^1118^* discs. The solid line represents the relative mean intensity of anti-BR-Z1 in *CSN4^null^* cells/neighboring *CSN4^null^/+* cells. The arrow marks the starting point when the BR-Z1 expression was significantly upregulated in *CSN4^null^* clones. Error bars represent the standard deviation (SD). Five *w^1118^* wing discs were scored (N = 5) for each time point, and at least three *CSN4^null^* wing discs were scored (N≥3) for each time point, except for 12–14 h APF (N = 2).

In response to ecdysone pulses, BR-Z1 expression is upregulated in wing-disc cells at late third-instar larval stages and peaked at the prepupal stage of 2–6 h APF (GFP-positive cells in Figure 4A–B′) [48]. BR-Z1 expression declined starting around 6–8 h APF and continued declining to almost undetectable level 24–28 h APF (GFP-positive cells in Figure 4C–F′). Quantification of immunofluorescent intensity following co-staining revealed that the BR-Z1 intensity was 1.6-fold of H3 intensity 2–6 h APF, but dramatically dropped to 0.07-fold of H3 24–28 h APF (dashed line in Figure 4G, and Figure S4A–B″), with the strongest reduction occurring between 12–14 h to 16–18 h APF.

Strikingly, we found that the CSN initiates BR-Z1 suppression during this time period. From the late third-instar larval stage to 8 h APF, the BR-Z1 levels in *CSN4^null^* cells were comparable to neighboring heterozygous *+/CSN4^null^* cells, suggesting that CSN4 has no role in regulating BR-Z1 levels at these stages (Figure 4A–C′, solid line in 4G). However, while BR-Z1 continued to decline at the beginning of the prepupa-to-pupa transition, higher BR-Z1 levels in *CSN4^null^* clones were detected. Significant BR-Z1 upregulation in *CSN4^null^* cells was first detected 14–16 h APF (Figure 4D, 4D′, and arrow and solid line in 4G), two hours after the completion of prepupal development. The upregulation in *CSN4^null^* cells reached about two-fold of neighboring heterozygous cells 14–18 h APF and three-fold 18–20 APF, and the peak of a 10-fold increase was observed 24–28 h APF (Figure 4E–F′, solid line in 4G). The enhancement of BR-Z1 expression in *CSN4^null^* cells declined after 28 h APF (solid line in Figure 4G).

**Figure 5:**
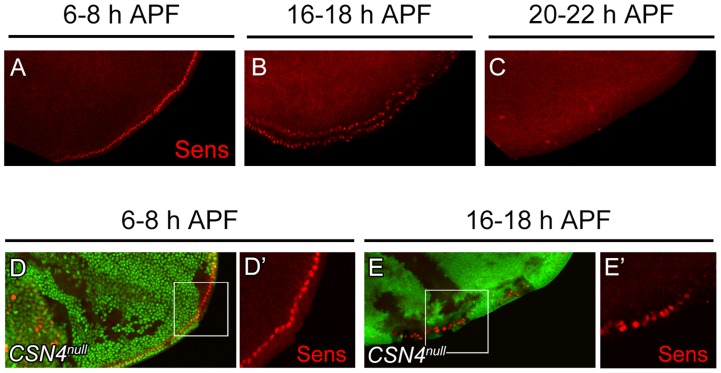
Temporal regulation of Sens expression by CSN4. (A–C) Sens (red) expression in wild-type wing discs at the PWM. The expression gradually declined from 6–8 h APF (A) to 16–18 h APF (B), and was below detection 20–22 h APF (C). (D–E′) Sens (red) levels at the PWM 6–8 h APF were identical between *CSN4^null^* cells and neighboring *CSN4^null^/+* cells (D, D′), and were upregulated in *CSN4^null^* clones 16–18 h APF (E, E′).

The activation of BR-Z1 expression in *CSN* mutants at pupal stages prefigures the timing in the upregulation of Sens. At the PWM, Sens was highly expressed in the precursors 6–8 h APF (Figure 5A) [4], declined to lower levels in lineage cells 16–18 h APF (Figure 5B), and became undetectable 20 h APF (Figure 5C). In *CSN4^null^* cells, while the Sens levels remained unchanged compared to neighboring control cells 6–8 h APF (Figure 5D, 5D′), Sens was upregulated at the pupal stage of 16–18 h APF (Figure 5E, 5E′), and the upregulation was maintained even 20–24 h APF (Figure 2C, 2C′). Taken together, the CSN downregulates BR-Z1 and Sens levels in a time-dependent manner, detected only after the prepupa-to-pupa transition.

### Ecdysone receptor represses BR-Z1 after the prepupa-to-pupa transition

**Figure 6:**
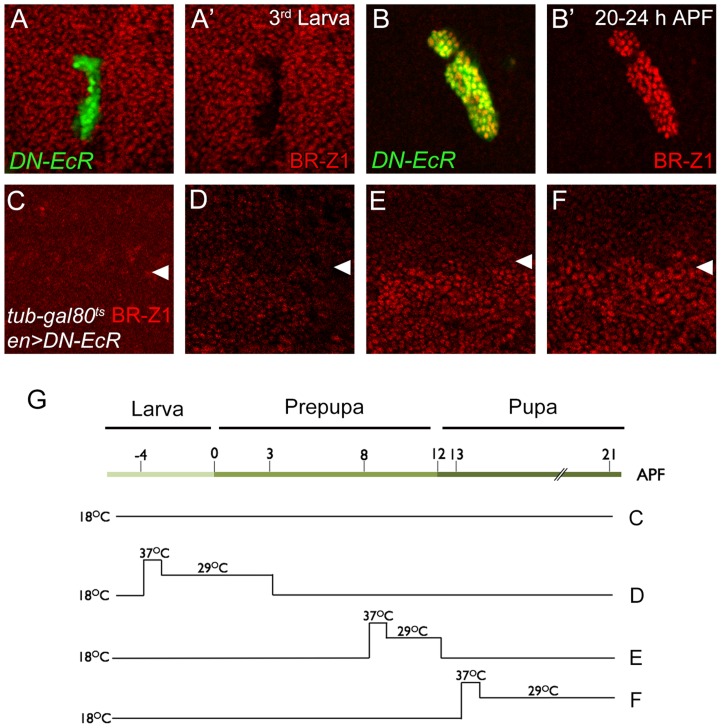
EcR regulation of BR-Z1 before and after the prepupa-to-pupa transition. (A–B′) DN-EcR-expressing clones (green) displayed BR-Z1 (red) downregulation at third-instar larval stages (A, A′) and upregulation 20–24 h APF (B, B′). (C–F) BR-Z1 (red) expression detected in wing discs of *en-GAL4>UAS-DN-EcR; Tub-GAL80^ts^/+* 21–22 h APF. White arrowheads indicate anterior-posterior boundaries. BR-Z1 expression in the posterior compartment (bottom) was unaffected when animals were incubated at 18°C from larva to pupa (C), or at 18°C except during larva-to-prepupa transition (−4 to 3 h APF) at non-permissive temperatures (37°C for one hour and 29°C for six hours) (D). BR-Z1 levels were upregulated in pupae incubated at non-permissive temperatures 8–12 h APF (E) or 13–21 h APF (F). (G) A diagram showing the temperature regimen in animals shown in Figure C–F.

To test whether ecdysone receptor activity is also involved in the switch from CSN-resistant to CSN-sensitive BR-Z1 suppression after the prepupa-to-pupa transition, the transcriptional activity of EcR was inactivated by the expression of dominant-negative *EcRB1* ^Δ*C655F645A*^ (DN-EcR) [49]. In DN-EcR expression clones, BR-Z1 expression in late larval wing discs was abolished (Figure 6A, 6A′), indicating that EcR positively regulates BR-Z1 expression [50]. In contrast, BR-Z1 expression was strongly induced in *DN-EcR* clones 20–24 h APF (Figure 6B, 6B′), indicating that transcriptionally active EcR represses BR-Z1 expression after the prepupa-to-pupa transition.

To further delineate the temporal regulation of BR-Z1 from activation to repression, DN-EcR was overexpressed by *en-GAL4* in the posterior compartment, in combination with temperature-sensitive *Tub-GAL80^ts^*. At the permissive temperature throughout larval to pupal stages, *en-GAL4*-driven DN-EcR expression was blocked by GAL80, and BR-Z1 remained at low levels in both anterior and posterior compartments (Figure 6C, 6G). At the non-permissive temperature, inactivation of GAL80 allowed expression of DN-EcR in the posterior compartment. We found that expression of DN-EcR from 4 h before puparium formation to 3 h APF maintained BR-Z1 repression in pupal wing discs at 21 h APF (Figure 6D, 6G). However, the DN-EcR expression at 8–12 or 13–21 h APF induced BR-Z1 upregulation in the posterior compartment (Figure 6E, 6F, 6G). Thus, active EcR is required to suppress BR-Z1 expression in pupal wings at and after the prepupa-to-pupa transition.

### CSN subunits associate with EcR to repress *br* transcription

**Figure 7:**
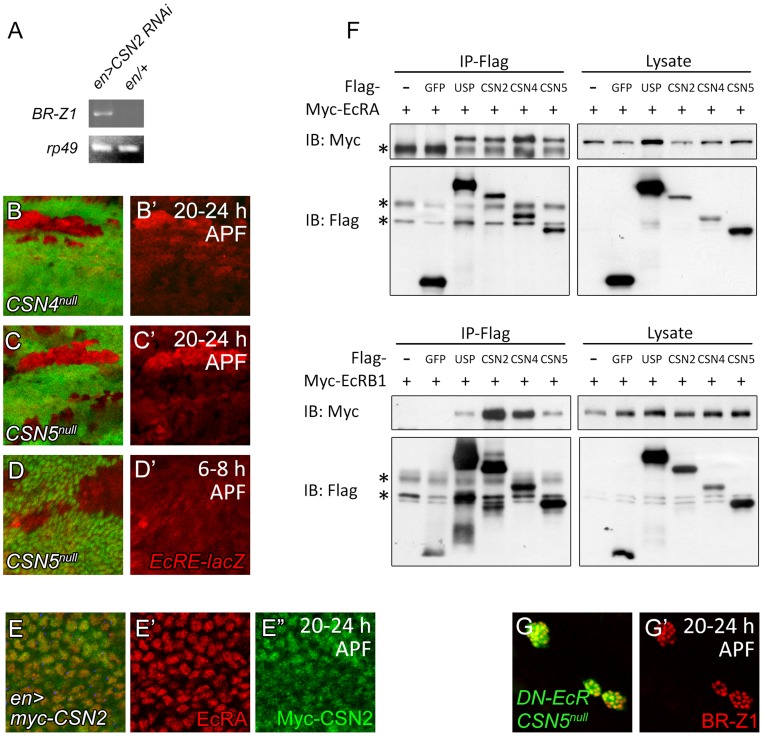
Association of CSN subunits and EcR in BR-Z1 repression. (A) The BR-Z1 mRNA level in wing discs 20–24 h APF, assayed by semi-quantitative RT-PCR, was upregulated in wing discs of *CSN2* RNAi driven by *en-GAL4* compared to *en-GAL4/+* control. *rp49* levels served as internal controls. (B–C′) *EcRE-lacZ* (red) expression was upregulated in *CSN4^null^* (B, B′) and *CSN5^null^* (C, C′) clones 20–24 h APF, but unaffected in *CSN5^null^* clones 6–8 h APF (D, D′). (E–E″) Both EcRA (red) and Myc-CSN2 (green) localized in nuclei in wing disc 20–24 h APF. (F) Western blots showing co-precipitations of Myc-EcRA (upper panel) or Myc-EcRB1 (lower panel) in Flag immunoprecipitates of Flag-USP, CSN2, CSN4 or CSN5 in S2 cell extract. Co-precipitation was not detected in Flag-GFP. * represents the non-specific bands. (G, G′) BR-Z1 levels (red) in double MARCM clones for *CSN5^null^* and *DN-EcR* (green) were not further elevated compared to *CSN5^null^* or *DN-EcR* single clones (Figure 3B, 6B).

EcR regulates BR-Z1 at the transcriptional level. To test whether the CSN also regulates BR-Z1 through transcriptional regulation, we measured the mRNA level for BR-Z1 in *CSN* mutants by semi-quantitative RT-PCR. While the BR-Z1 mRNA expression in *en-GAL4* control pupal wing discs was undetected, expression of *CSN2* RNAi by *en-GAL4* induced the BR-Z1 mRNA level (Figure 7A). To test the transcriptional regulation by the CSN, the reporter *EcRE-LacZ* which contains seven tandem repeats of *EcRE* was used [51]. In *CSN4^null^* and *CSN5^null^* clones, the expression levels from *EcRE-lacZ* were also elevated in comparison to neighboring heterozygous cells in pupal wings (Figure 7B–C′). The regulation of *EcRE-lacZ* is specific to the pupal stage, as *EcRE-LacZ* expression was not elevated in *CSN5^null^* clones at the prepupal stage (Figure 7D, 7D′). Thus, the CSN-downregulated BR-Z1 expression is at the transcriptional level and after the prepupa-to-pupa transition.

As a transcriptional regulator, EcRA was localized in nuclei in wing-disc cells (Figure 7E, 7E′). Examination of protein location of the Myc-tagged CSN2 and CSN4 subunits and the endogenous CSN5 subunit in wing disc cells showed that they primarily localized in nuclei as EcRA (Figure 7E, 7E″ and Figure S5A–B″). Thus, several CSN subunits and EcRA colocalize in nuclei, consistent with their roles in transcription regulation of BR-Z1.

We then performed co-immunoprecipitation to detect protein-protein interaction between CSN subunits and EcR. The S2 cells were co-transfected with expression plasmids for one of Flag-tagged CSN subunits and Myc-tagged EcRA or EcRB1. Both EcRA and EcRB1 were specifically detected in the Flag immunoprecipitates of CSN2, CSN4, CSN5 and the positive control USP, but not the negative control GFP (Figure 7F). The abilities of various subunits to associate with EcR suggest that the CSN interacts with EcR as protein complexes.

The results show that inactivating either the CSN or EcR is sufficient to derepress BR-Z1 expression at the pupal stage (Figure 3A–B′, 6B). We then examined the effect on BR-Z1 expression when both CSN and EcR were inactivated. In double mutants that expressed DN-EcR in *CSN5^null^* mutant clones, BR-Z1 was upregulated to the level comparable to that in single mutants of *CSN5^null^* or DN-EcR (Figure 7G, 7G′). This result supports the notion that the CSN and EcR function as protein complexes to repress BR-Z1 expression.

### Nedd8 and multiple cullins are required to suppress BR-Z1 and Sens expressions

**Figure 8:**
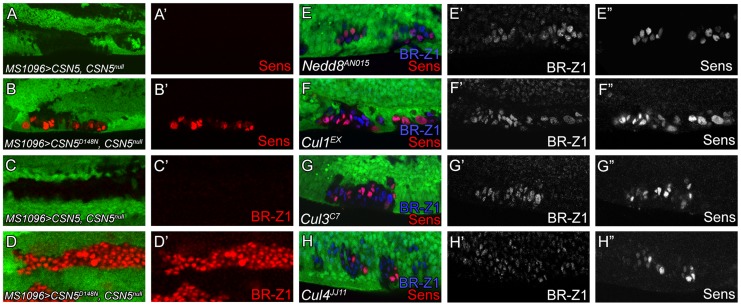
Deneddylation/neddylation and multiple cullins suppress BR-Z1 and Sens expressions at the PWM. (A–H″) 20–24 h APF wing discs. (A–D′) Upregulation of Sens (red in A–B′) and BR-Z1 (red in C–D′) in PWM *CSN5^null^* clones were suppressed by wild-type CSN5 (A, A′, C, C′), but not deneddylation-defective CSN5^D148N^ (B, B′, D, D′). (E–H) Upregulation of BR-Z1 (blue) and Sens (red) were observed in null clones of *Nedd8^AN015^* (E), *Cu11^EX^* (F), *Cul3^C7^* (G) and *Cul4^JJ11^* (H) at the PWM 20–24 h APF, with a single channel for BR-Z1 (E′–H′) and Sens (E″–H″).

Deneddylation is coupled with neddylation to cycle cullins between two neddylation states for optimal CRL activities [52]. We first addressed whether the deneddylating activity of the CSN is required to repress BR-Z1 and Sens. Elevated BR-Z1 and Sens expressions in *CSN5^null^* cells were completely suppressed by the wild-type *CSN5* transgene driven by *MS1096-GAL4* in wing discs (Figure 8A, 8A′ 8C, 8C′). However, expression of the deneddylation-defective *CSN5^D148N^* mutant [26] failed to decrease BR-Z1 and Sens levels in *CSN5^null^* clones (Figure 8B, 8B′, 8D, 8D′). This result suggests the Nedd8 conjugation is involved in BR-Z1 and Sens regulations. Indeed, *Nedd8^AN015^* null mutant clones located at the PWM of pupal wings also exhibited BR-Z1 and Sens upregulations (Figure 8E–E″).

We then addressed whether and which cullins are affected in *CSN* mutants for control of Sens and BR-Z1 suppression. Individual cullin proteins were inactivated by generating clones for the null alleles *Cul1^EX^*, *Cul3^C7^* and *Cul4^JJ11^*. Interestingly, these *Cul* mutants showed upregulations of BR-Z1 and Sens in pupal wing discs of 20–24 h APF (Figure 8F–H″). Consistent with the pupa-specific regulation, BR-Z1 levels remained unchanged in the *Cul1^EX^*, *Cul3^C7^* and *Cul4^JJ11^* clones of late third instar larval wing discs (Figure S6A–C″). Therefore, our results indicate that cycling multiple cullin components between neddylation and deneddylation is required to completely suppress BR-Z1 and Sens in pupal PWM cells.

## Discussion

To build the nervous system, spatial and temporal patterning cues have to be integrated to confer neural competence at specific locations of the developing tissues. We describe a critical role of the CSN, which by reading the temporal ecdysone signal cue, can determine neural competence at a specific location, the PWM. Interestingly, in response to ecdysone signaling, EcR activity is converted from transcription activation to repression of target genes, and the CSN-mediated regulation is involved in this conversion. Repression of the target gene *br* leads to the repression of BR-Z1-activated Sens, thus suppressing the neural differentiation of PWM bristles. We show that the CSN physically associates with EcR and represses BR-Z1 at the transcriptional level. Together with the genetic analyses between CSN subunits and EcR, our study indicates that the CSN integrates the temporal hormone signaling to endow a spatial neural repressive activity in sensory bristle patterning.

### Suppression of neural competence of non-innervated bristle at the PWM

The difference between AWM and PWM bristles is that AWM bristles are innervated by sensory neurons to sense mechanical or chemical stimulations. PWM bristles with long and thin hair structures are non-innervated and without socket support, and they are non-functional in sense detection. During development, the transcription factor Sens is expressed dynamically at the wing margin. At the third-instar larval stage, Sens is initially expressed in both anterior and posterior margins. Expressions of proneural proteins Ac and Sc, which are limited to anterior cells, maintain Sens in developing chemosensory bristle cells [4]. At 6–8 h APF, Sens is re-activated in precursors of both anterior mechanosensory and posterior non-innervated bristles for precursor specification. At the pupal stage, however, Sens expression is suppressed in bristle lineage cells, thus preventing neural differentiation at PWM. Timely suppression of Sens expression plays a critical role in disruption of neural differentiation of PWM bristles, as continuing Sens expression allows PWM bristles to differentiate into innervated bristles, even after precursor specification.

While *sens* knockdown in bristle lineage cells prevented transformation of PWM non-innervated to innervated bristles in *CSN* mutants, it did not transform innervated bristles to non-innervated bristles at AWM (Figure S7A, S7B), indicating that diminishment of Sens levels in the lineage cells is not the sole determinant in non-innervated bristle formation, and additional mechanisms are activated to disrupt the differentiation of neurons and the associated neural cells at the PWM. It is shown that programmed cell death of the underlying neurons and sheath cells is one such mechanism. Suppression of programmed cell death, either by overexpression of anti-apoptotic protein p35 or by blocking Dmyb and Grim activities, induces ectopic neurons associated with PWM bristles [4], [53]. While overexpression of p35 prevented neuronal death (Figure S8B), we found that the morphology of PWM hairs was not affected and lacked associated sockets (). Thus, apoptosis has an effect only on internal neurons and sheath cells, while other mechanisms are required to prevent socket cell formation and to differentially regulate hair elongation at PWM.

### Switch from activation to repression of ecdysone signaling target genes requires CSN deneddylation activity

Pulses of ecdysone provide temporal information to coordinate various developmental processes in insect development. Activation of ecdysone signaling in late larval and prepupal stages are required for larva-to-prepupa and prepupa-to-pupa transitions, respectively. In contrast to *br* activation in the larva-to-prepupa transition, *br* expression was thought to be low even with a large ecdysone pulse in the pupal stage [10], [19]. Our study indicates that this is indeed due to repression of *br* by ecdysone signaling, as shown by the expressions of BR-Z1 induced by dominant-negative EcR in pupal wings. The repressive regulation requires the input of the CSN, as BR-Z1 was also upregulated in pupal wing-disc cells of *CSN4^null^* and *CSN5^null^* mutants. Therefore, our results suggest that by integrating the CSN, ecdysone signaling activity plays a non-canonical role in the prepupa-to-pupa transition, and the *br* repression is critical to Sens suppression during PWM bristle development.

Neddylation is required for activating CRL activity both *in vivo* and *in vitro* [52], [54]–[56]. Deneddylation, while inactivating CRL *in vitro*, promotes CRL activity *in vivo* by protecting them from cellular degradation, thus maintaining a physiological pool of CRL for the next round of neddylation [26], [27]. Our results showed that repression of ecdysone signaling target genes requires cycling of Nedd8 substrates between neddylation and deneddylation states. Similar to *CSN* mutants, the protein levels of BR-Z1 and Sens were elevated in *Nedd8* mutants. Also, CSN suppression of BR-Z1 and Sens depends on the deneddylating activity residing in the CSN5 subunit, as the deneddylation-defective CSN5 mutant failed to rescue the *CSN5* phenotype. Thus, the requirement of neddylation and deneddylation is consistent with the involvement of multiple cullin proteins that are the major substrates of Nedd8 conjugation. Transcription regulation of ecdysone responsive genes requires multiple activators and repressors [57]–[61] whose protein activity and stabilities could be in turn regulated by multiple cullin-organized ubiquitin E3 ligases. Alternatively, these cullin ligases might act on the same set of targets, like the regulations of protein stability of Ci by multiple cullin ligases [54].

Our results on the requirement of the CSN holoenzyme in development of PWM bristles suggest different usages of CSN subunits in nuclear receptor signaling. A previous study showed that CSN4 but not CSN5 is required for larval molting [32]. In mammals, single CSN2 and CSN5 subunits interact with nuclear receptors to function as a co-repressor and a co-activator, respectively, in steroid hormone signaling [62]–[64]. *Drosophila* CSN2 physically interacts with several *Drosophila* hormone receptors including EcR [62]. In our study, seven out of the eight CSN subunits were shown to be required for Sens suppression and CSN2, CSN4 and CSN5 were shown to associate with EcR, strongly suggesting that the CSN associates with EcR as a complex. It has been shown that the CSN complex interacts with multiple cullins to protect them from self-ubiquitination and degradation [26], [65]. Thus, the association between EcR and the CSN holoenzyme might provide a platform for recruiting and maintaining multiple CRLs, composed of cullins and their associated components, in the proximity for efficient down-regulation of EcR-mediated transcription (Figure S9). Two models are proposed here for the CSN-dependent switch of EcRs from activation to repression of *br* (Figure S9). In model A, the switch depends on the induction of specific co-repressors upon the pulse of ecdysone at the prepupa-to-pupa transition. The co-repressor could then replace the co-activator, a step that could be facilitated by the nearby CRLs through the ubiquitination of co-activators for subsequent degradation, for example. In model B, the switch depends on the activation of specific CRLs by ecdysone signaling at the prepupa-to-pupa transition. The activation could be mediated through expression of the specific substrate receptors of CRLs, or phosphorylation of substrates to induce binding to substrate receptors. Once activated, these CRLs could inactivate co-activators to shut down *br* transcription. It is unlikely that the CSN-mediated pupa-specific BR-Z1 and Sens repression is through mechanisms involving changes in the CSN and EcR protein expression levels during the prepupa-to-pupa transition, as the expression of the catalytic subunit CSN5 and EcRA were expressed at comparable levels in wing discs at late third instar larval and pupal stages (Figure S10A–C, and S11A–F′). Also, the CSN is active in third-instar larvae for normal wing disc development [37]. Our finding that suppression of EcR target genes in pupae through the CSN and multiple cullins provide insights for a novel negative regulation of steroid hormone signaling in metazoa.

## Materials and Methods

### Fly strains and genetics

The following fly strains were used in this study. Mutant alleles: *CSN5^null^*, *CSN4^null^* [32], *Nedd8^AN015^*, *Cul1^EX^* [54], *Cul3^C7^* [66], *Cul4^JJ11^* [67], *sc^10-1^* [68], *br^npr1^* [69], [70]. Reporter line: *EcRE-LacZ* (II, BL4516) [71]. GAL4 drivers: *C96-GAL4* [72], *en-GAL4* [73], *neur-GAL4* [74] and *MS1096-GAL4* [75]. UAS lines: *UAS-BR-Z1*, *UAS-BR-Z3* [76], *UAS-p35* (*P[UAS-p35.HB]H3*, BL6298) [77], *UAS-EcRB1* ^Δ*C655F645A*^ [49], *UAS-CSN5, UAS-CSN5^D148N^* [26], *UAS-myc-CSN2* (this study), *UAS-myc-CSN4* (this study), and *UAS-3Xflag-sens* (this study). The *UAS-CSN1bRNAi* (v34727), *UAS-CSN2RNAi* (v48044), *UAS-CSN3RNAi* (v101516), *UAS-CSN6RNAi* (v22308) and *UAS-CSN7RNAi* (v40691) were obtained from Vienna *Drosophila* RNAi Center (VDRC). The *UAS-sensRNAi* (NIG 32120R-2) was obtained from NIG-FLY stock center.

Clones were generated by FLP/FRT-mediated mitotic recombination [78]. The mutant or MARCM clones in developing tissues were identified by the absence or presence of GFP, respectively. For transient overexpression using GAL80^ts^, developing animals were grown at 18°C. Prepupae or pupae were then shifted to 37°C for one hour to rapidly inactivate GAL80^ts^ to allow fast GAL4-activated gene expression, and then incubated at 29°C for several hours as indicated.

### Immunofluorescence staining

Larvae and prepupae (0–10 h APF) were dissected in 1×PBS for isolating wing discs, which were then fixed in 4% paraformaldehyde for 15 minutes at room temperature (RT). For pupae 12–36 h APF, pupal cases were removed in 1×PBS and the whole pupae were fixed in 4% paraformaldehyde at RT for one hour. Internal pupal membranes on the surface of wing tissues were subsequently removed and the resulting wing discs were fixed again in 4% paraformaldehyde for 15 minutes. The following primary antibodies were used: mouse anti-Ac (DSHB, 1∶5) [79], mouse anti-ß-galactosidase 40-1a (DSHB, 1∶500) [80], mouse anti-BR-Z1 Z1.3C11.OA1 (DSHB, 1∶250) [48], mouse anti-EcRA 15G1a (DSHB, 1∶100) [81], mouse anti-Elav 9F8A9 (DSHB, 1∶500) [82], mouse anti-Futsch 22C10 (DSHB, 1∶500) [83], mouse anti-Hnt 1G9 (DSHB, 1∶25) [84], mouse anti-Pros MR1A (DSHB, 1∶100) [85], rabbit anti-c-Myc A-14 (Santa Cruz, 1∶500), mouse anti-Pol II CTD4H8 (Santa Cruz, 1∶500), rabbit anti-Histone H3 GTX122148 (GeneTex, 1∶500), rabbit anti-JAB1 (CSN5) (Sigma, 1∶250), and guinea pig anti-Sens (1∶2000) [3]. The anti-JAB1/CSN5 antibody was pre-cleaned by incubation of 10× volume antibody with *CSN5^null^* larval brain tissues at 4°C overnight.

### Quantification of staining intensity

For assaying anti-BR-Z1 and anti-histone H3 immunostaining intensity, all images were obtained from the Zeiss LSM 510 Meta microscope with the same setting, and analyzed by Image J. For comparing BR-Z1 intensity in *CSN4^null^* clones and *CSN4^null^/+* cells, the mean nuclear anti-BR-Z1 intensity per pixel in each wing disc was calculated from twenty randomly selected cells within *CSN4^null^* clones or the neighboring heterozygous tissue. For comparing BR-Z1 and histone H3 intensity, the mean intensity per pixel was obtained from the same twenty randomly selected cells in each *w^1118^* wing discs. The mean intensity of CSN5 and Pol II in *w^1118^* wing discs was measured using the same method.

### Cell culture and plasmid construction

S2 cells were maintained in Schneider medium (Invitrogen) supplemented with 10% fetal bovine serum. Cells were transiently transfected with UAS-based expression plasmids together with driver *pWA-GAL4* [86]. Transfection was carried out using Cellfectin II reagent (Invitrogen). The expression plasmids for CSN2, 4, 5, EcRA, and EcRB1 were constructed by cloning the ORFs into *Drosophila* gateway vectors pTFW or pTMW (obtained from Drosophila Genomics Resource Center, DGRC). The *UAS-3Xflag-sens* plasmid was constructed by cloning the 3Xflag-sens DNA into the *pUAST* vector.

### Co-immunoprecipitation

The harvested cells were washed twice with ice-cold 1×PBS, and then lysed in mRIPA buffer [50 mM Tris-HCl (pH 7.8), 150 mM NaCl, 5 mM EDTA (pH 8.0), 0.5% Triton X-100, 0.5% NP-40] supplemented with complete protease inhibitors (Roche). Lysates were diluted in mRIPA buffer to a final concentration of 5 ug/ul. 20 ul ANTI-FLAG M2 Affinity Gel (Sigma) was added to 1 mL lysate and incubated for overnight at 4°C. Associated protein complexes were analyzed by SDS-PAGE followed by western blotting. The primary antibodies used for western blots were mouse anti-Flag M2 (Sigma, 1∶10000) and mouse anti-c-Myc 9E10 (Santa Cruz, 1∶5000). Secondary antibody was goat anti-mouse HRP used at 1∶5000.

### RT-PCR

Pupal wing discs were dissected in ice-cold 1×PBS. Total mRNA from thirty to forty pairs of pupal wing discs were extracted by TRIzol RNA Isolation Reagents (Invitrogen) and reverse-transcribed to cDNA by MMLV RT (PROSPEC). Expression of BR-Z1 mRNA was detected by the primer set: 5′—TGAAG AGGAG TGGTG ATTGA GCTGC—3′ and 5′—CCATC ACAAG TGCCT CCGGC ATC—3′. The mRNA for *rp49* was used as the internal control.

## Supporting Information

Figure S1 Ectopic sensory bristle formation at PWM of *CSN1b* and *CSN7* knockdown flies and Hnt expression pattern in wild-type and *CSN5* mutant wing discs. (A, B) PWM bristles of *CSN1b* RNAi (A) and *CSN7* RNAi (B) knockdown by *en-GAL4*. Arrowheads indicate single bristles with a thicker shaft and a socket. Arrows indicate single bristles with two shafts and one large socket. (C) Hnt (red) was expressed at high levels at the AWM (C, C′) and low levels at the PWM (C, C″) in wild-type disc 20–24 h APF. Arrowhead indicates the anterior-posterior boundary. (D, D′) Hnt (red) expression was elevated in *CSN5* clones at PWM 20–24 h APF.
(TIF)

Figure S2 Sens and Ac expression patterns in *CSN* mutants. (A, B) Sens (red) expression was upregulated at the PWM of *CSN2* RNAi (A) and *CSN3* RNAi (B) wing discs 20–24 h APF driven by *en-GAL4*. (C, D) Upregulation of Sens (red) at the PWM of *CSN1b RNAi CSN7 RNAi* knockdown wing discs 24–26 h APF by *neur-GAL4* (C) was strongly reduced when *sens* was simultaneously knockdown (D). (E–F′) Ac (red) expression was not induced in *CSN5^null^* clones at the PWM of late third instar larva (E, E′) and 4–8 h APF prepupa (F, F′). Arrowheads indicate the anterior-posterior boundary.
(TIF)

Figure S3 Induction of Sens and innervated bristles at the PWM by BR-Z3 overexpression. (A) Knockdown of *CSN2* by wing margin *C96-GAL4* upregulated BR-Z1 expression at the wing margin 20–24 h APF. (B, C) BR-Z3 overexpression, driven by *C96-GAL4*, induced ectopic Sens (red) expression at the PWM 20–24 h APF (B), and ectopic innervated bristles with sockets (arrows) at the adult PWM (C).
(TIF)

Figure S4 Anti-BR-Z1 and co-stained anti-histone H3 staining at late larval and pupal stages. (A–B″) BR-Z1 (red) and histone H3 (green) immunostaining in wild type *w^1118^* wing discs 2–6 h APF (A–A″) and 24–28 h APF (B–B″). By comparing to H3 staining, BR-Z1 expression was at the highest level 2–6 h APF and was repressed to a non-detectable level 24–28 h APF.
(TIF)

Figure S5 Nuclear localization of CSN4 and CSN5 protein in wing discs. (A–A″) Myc-CSN4 (green), expressed by *C96-GAL4*, localized in the nucleus as well as in cytoplasm in late larval wing disc cells (A and A″). EcRA localized in the nucleus (A′). (B–B″) CSN5 (green) detected by the anti-JAB1/CSN5 antibody primarily localized in the nucleus in late larval wing disc cells, as suggested by the co-localization with the nuclear GFP (nGFP) (red). The anti-JAB1/CSN5 antibody specifically recognized endogenous CSN5, as shown by strongly reduced immunofluorescent intensity in the *CSN5^null^* clones (marked by the absence of nGFP in B′).
(TIF)

Figure S6 BR-Z1 expression is not affected in cullin mutants at late third instar larval stage. (A–C″) BR-Z1 (red) expression in *Cul1^EX^*, *Cul3^C7^* and *Cul4^JJ11^* clones was comparable to that in the neighboring heterozygous cells in late third instar larval wing discs.
(TIF)

Figure S7 Reduction of Sens in bristle lineage cells does not affect innervated bristle formation at AWM. (A) High-level Hnt (red) was observed at AWM of *sens* knockdown wing disc 20–24 h APF by *neur-Gal4*. (B) Morphologically normal AWM bristles with sockets were observed at AWM of *sens* knockdown adult wing by *neur-Gal4*.
(TIF)

Figure S8 Inhibition of apoptosis does not affect the morphology of PWM bristles. (A) Neurons labeled by anti-Elav antibody (red) were detected at anterior but not posterior wing margin in control *en-GAL4* wing disc 20–24 h APF. (B–D) Overexpression of anti-apoptotic protein p35 in the posterior compartment of the wing discs by *en-GAL4*. (B) Overexpression of p35 induced neurons (red) at PWM of 20–24 h APF wing disc. (C) Adult wing of p35 overexpression by *en-GAL4*. (D) Enlargement of the marked area in (C), showing formation of the morphologically normal PWM bristles with thin, long hair without socket support.
(TIF)

Figure S9 Proposed models for CSN-dependent switch of ecdysone receptor from activation to repression. We propose that the CSN, together with the cullin-based CRLs, is recruited to the *br* gene locus through association with EcR. While associated with EcR constitutively, the CSN-dependent pupa-specific switch of EcR to repressor may utilize two mechanisms: (A) the switch is mediated by induction of the specific co-repressor of EcR upon activation of ecdysone signaling at the prepupa-to-pupa transition. The co-repressor could replace the co-activator, a step facilitated by the nearby CRLs through a mechanism such as ubiquitination of co-activators for subsequent degradation. (B) The EcR switches from the activator to repressor through specific CRLs activated by ecdysone signaling at the prepupa-to-pupa transition. The CRL activation could be mediated through pupa-specific expression of substrate receptors or phosphorylation of the substrates to induce binding to the CRLs. These activated CRLs shut down *br* transcription through a mechanism such as inactivation of co-activators. Please see Discussion for more explanation of these models.
(TIF)

Figure S10 CSN5 is constitutively expressed in wing discs at late larval and pupal stages. (A–B′) CSN5 (red) detected by the anti-JAB1/CSN5 antibody was ubiquitously expressed in wild-type *w^1118^* wing discs at late third instar larva (A, A′) and 16–18 h APF (B, B′). The co-stained anti-Pol II antibody staining (green) was used as a control for comparison of staining intensity in discs from different stages. (C) Diagram of the mean relative intensities of anti-CSN5 vs. co-stained anti-Pol II at late instar larva and 16–18 h APF. Five (N = 5) *w^1118^* wing discs were scored at both time points.
(TIF)

Figure S11 CSN does not regulate EcRA expression. (A–F′) EcRA (red) is constitutively expressed from late third instar larval to pupal stages, and its levels remained constant in *CSN4^null^* clones compared to the neighboring *CSN4^null^/+* cells from late third larval instar to pupa.
(TIF)
