# Supplementary figures and images for: The COP9 Signalosome Converts Temporal Hormone Signaling to Spatial Restriction on Neural Competence
Source: PLoS Genet. 2014 Nov 13;10(11):e1004760. doi: 10.1371/journal.pgen.1004760 (PMC4230841; doi:10.1371/journal.pgen.1004760)

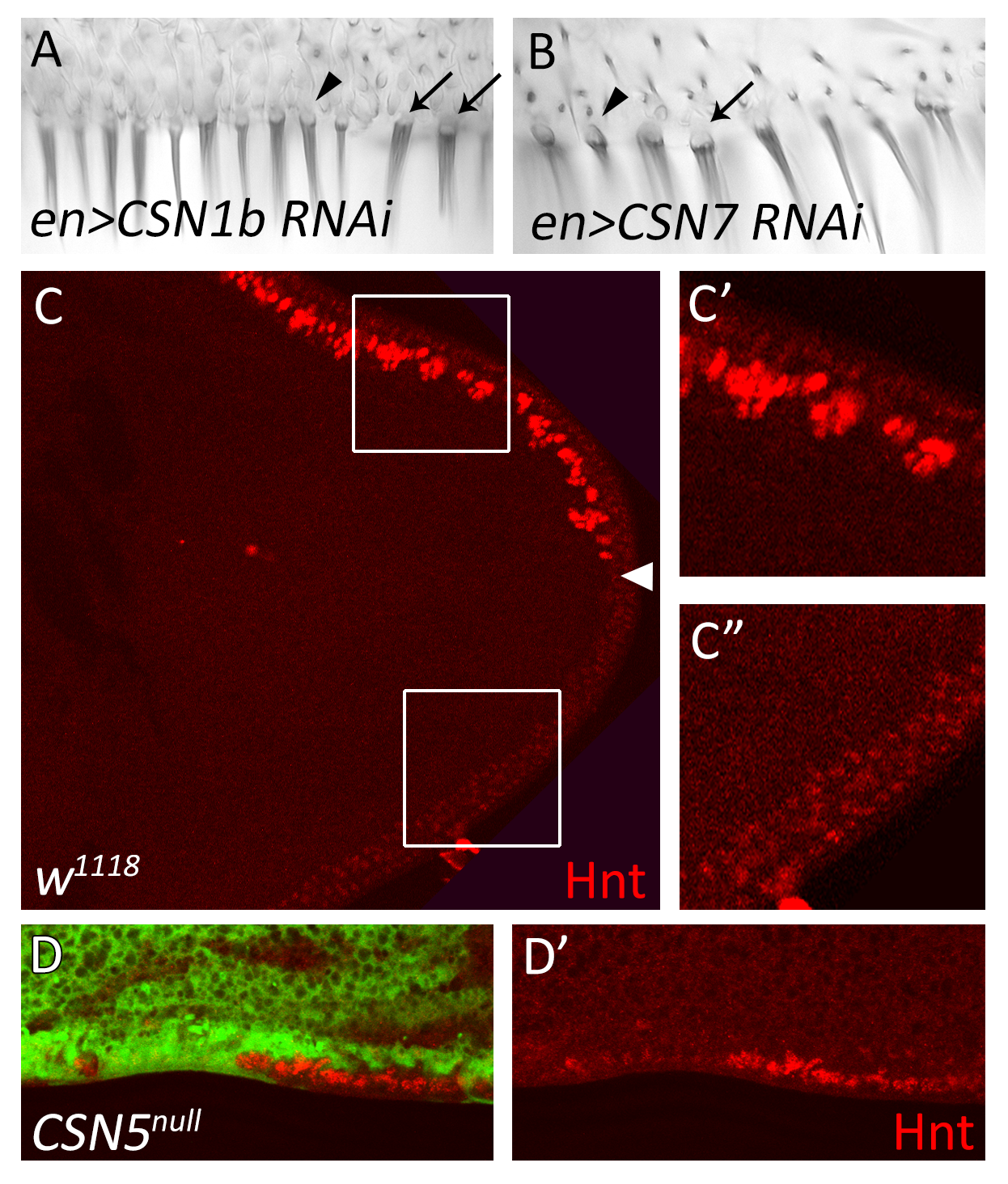

Supplement: Figure S1 — Ectopic sensory bristle formation at PWM of CSN1b and CSN7 knockdown flies and Hnt expression pattern in wild-type and CSN5 mutant wing discs. (A, B) PWM bristles of CSN1b RNAi (A) and CSN7 RNAi (B) knockdown by en-GAL4. Arrowheads indicate single bristles with a thicker shaft and a socket. Arrows indicate single bristles with two shafts and one large socket. (C) Hnt (red) was expressed at high levels at the AWM (C, C′) and low levels at the PWM (C, C″) in wild-type disc 20–24 h APF. Arrowhead indicates the anterior-posterior boundary. (D, D′) Hnt (red) expression was elevated in CSN5 clones at PWM 20–24 h APF. (TIF) [file pgen.1004760.s001.tif]

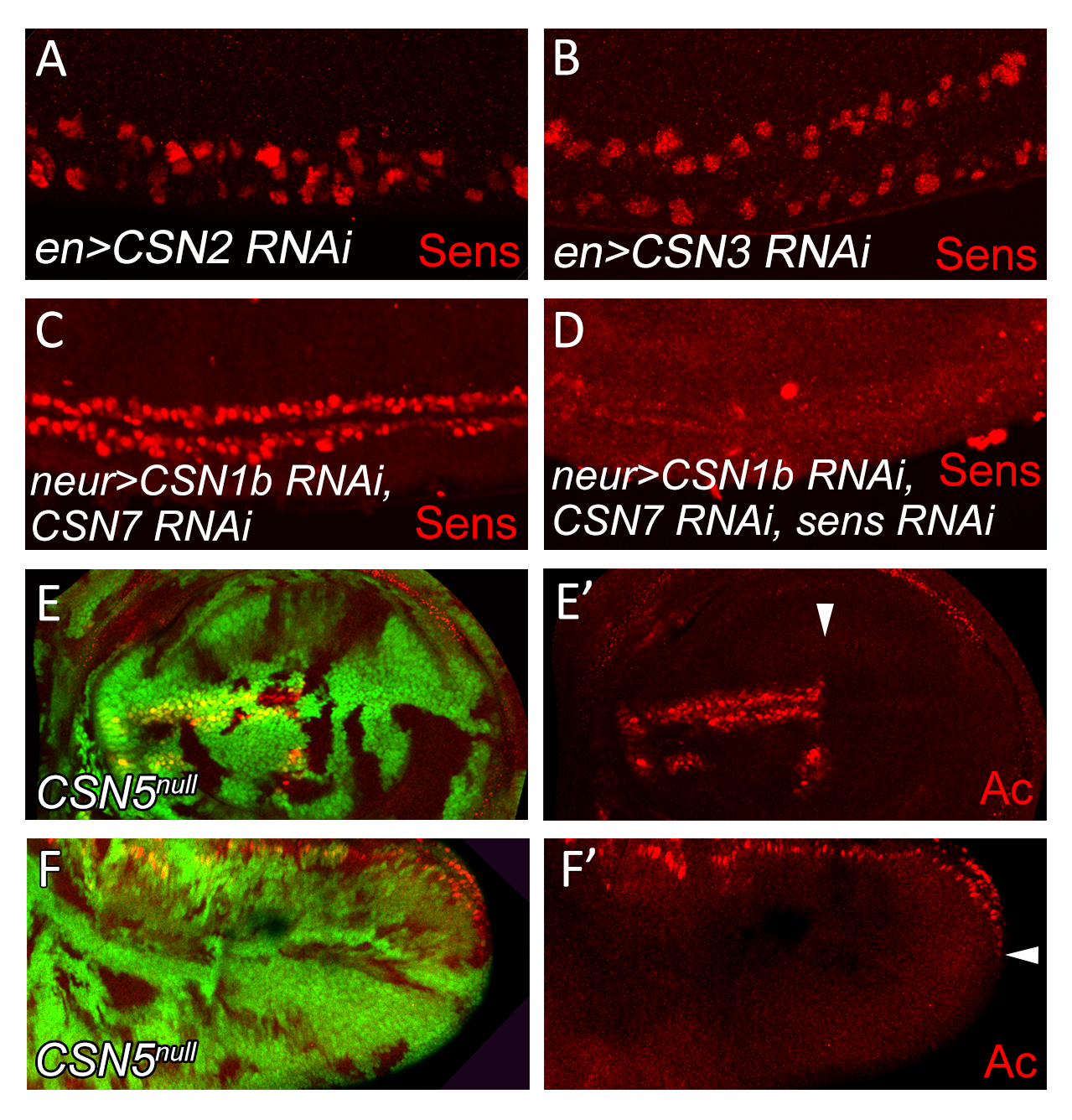

Supplement: Figure S2 — Sens and Ac expression patterns in CSN mutants. (A, B) Sens (red) expression was upregulated at the PWM of CSN2 RNAi (A) and CSN3 RNAi (B) wing discs 20–24 h APF driven by en-GAL4. (C, D) Upregulation of Sens (red) at the PWM of CSN1b RNAi CSN7 RNAi knockdown wing discs 24–26 h APF by neur-GAL4 (C) was strongly reduced when sens was simultaneously knockdown (D). (E–F′) Ac (red) expression was not induced in CSN5null clones at the PWM of late third instar larva (E, E′) and 4–8 h APF prepupa (F, F′). Arrowheads indicate the anterior-posterior boundary. (TIF) [file pgen.1004760.s002.tif]

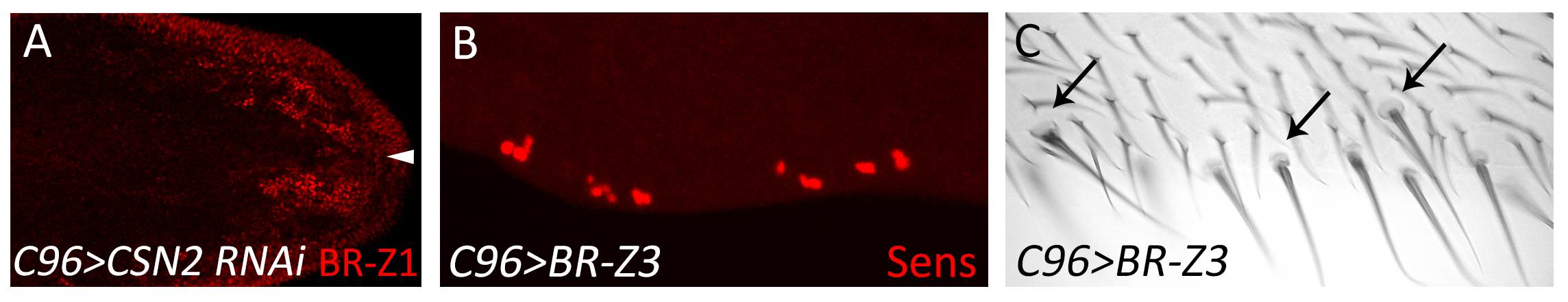

Supplement: Figure S3 — Induction of Sens and innervated bristles at the PWM by BR-Z3 overexpression. (A) Knockdown of CSN2 by wing margin C96-GAL4 upregulated BR-Z1 expression at the wing margin 20–24 h APF. (B, C) BR-Z3 overexpression, driven by C96-GAL4, induced ectopic Sens (red) expression at the PWM 20–24 h APF (B), and ectopic innervated bristles with sockets (arrows) at the adult PWM (C). (TIF) [file pgen.1004760.s003.tif]

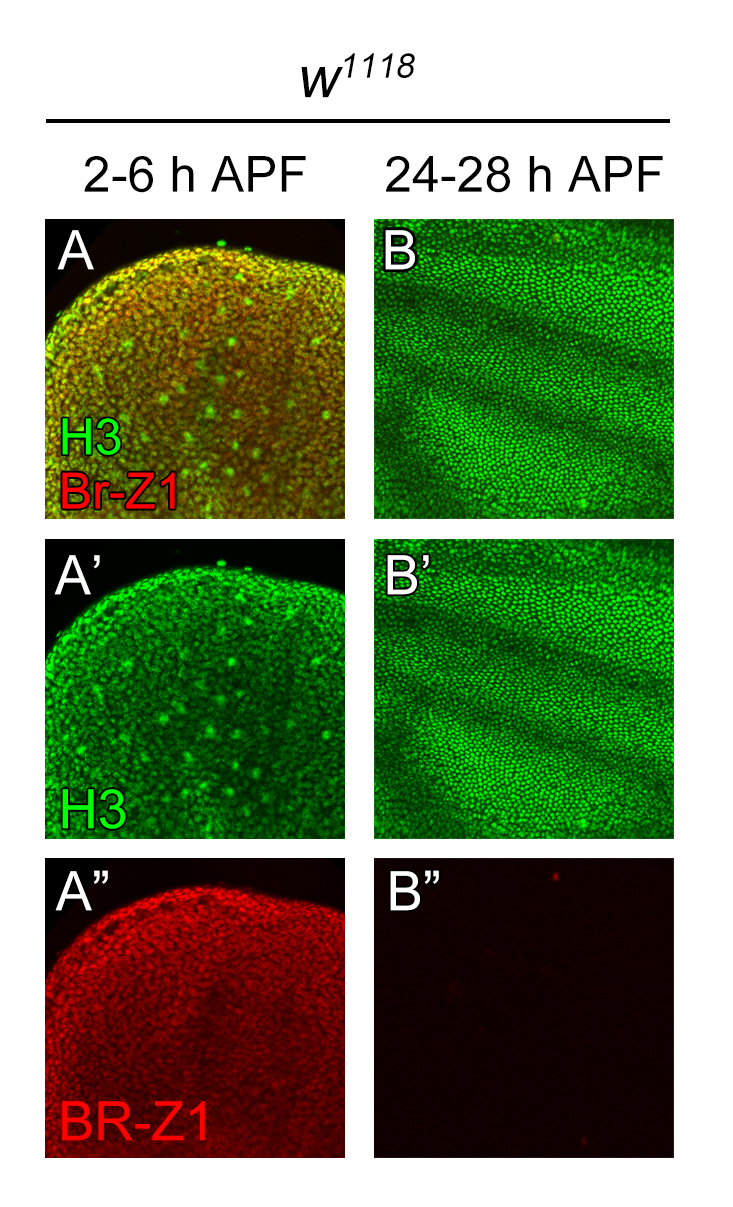

Supplement: Figure S4 — Anti-BR-Z1 and co-stained anti-histone H3 staining at late larval and pupal stages. (A–B″) BR-Z1 (red) and histone H3 (green) immunostaining in wild type w1118 wing discs 2–6 h APF (A–A″) and 24–28 h APF (B–B″). By comparing to H3 staining, BR-Z1 expression was at the highest level 2–6 h APF and was repressed to a non-detectable level 24–28 h APF. (TIF) [file pgen.1004760.s004.tif]

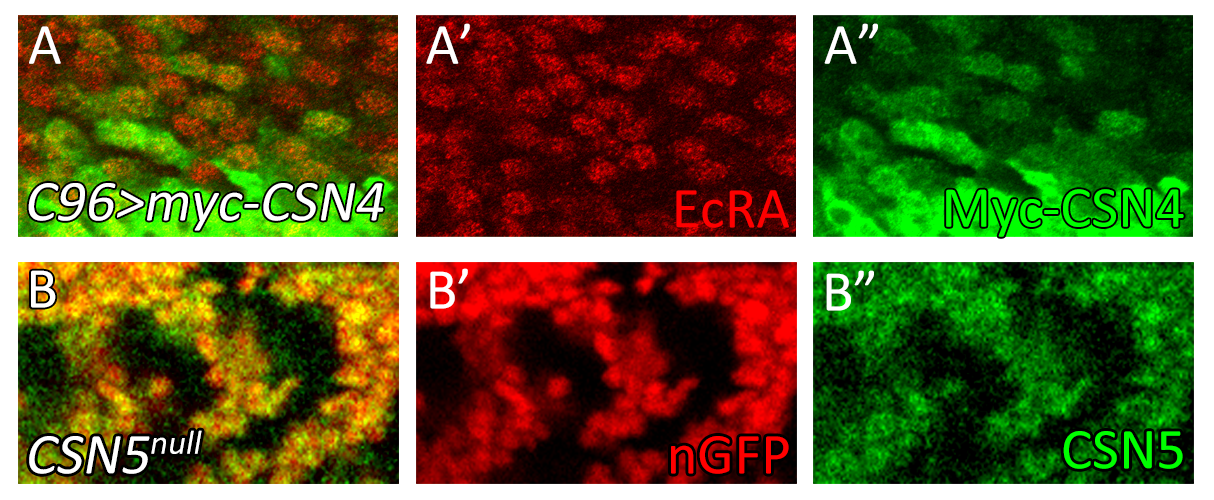

Supplement: Figure S5 — Nuclear localization of CSN4 and CSN5 protein in wing discs. (A–A″) Myc-CSN4 (green), expressed by C96-GAL4, localized in the nucleus as well as in cytoplasm in late larval wing disc cells (A and A″). EcRA localized in the nucleus (A′). (B–B″) CSN5 (green) detected by the anti-JAB1/CSN5 antibody primarily localized in the nucleus in late larval wing disc cells, as suggested by the co-localization with the nuclear GFP (nGFP) (red). The anti-JAB1/CSN5 antibody specifically recognized endogenous CSN5, as shown by strongly reduced immunofluorescent intensity in the CSN5null clones (marked by the absence of nGFP in B′). (TIF) [file pgen.1004760.s005.tif]

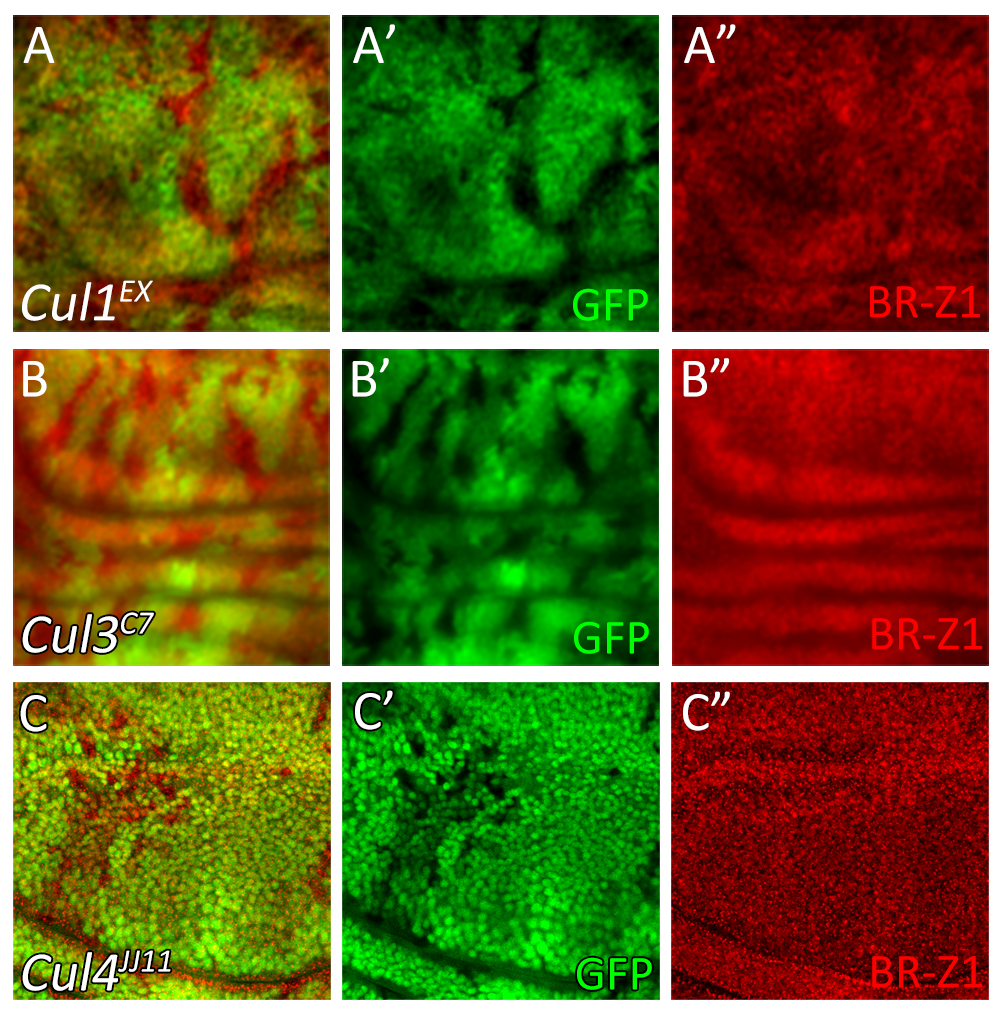

Supplement: Figure S6 — BR-Z1 expression is not affected in cullin mutants at late third instar larval stage. (A–C″) BR-Z1 (red) expression in Cul1EX, Cul3C7 and Cul4JJ11 clones was comparable to that in the neighboring heterozygous cells in late third instar larval wing discs. (TIF) [file pgen.1004760.s006.tif]

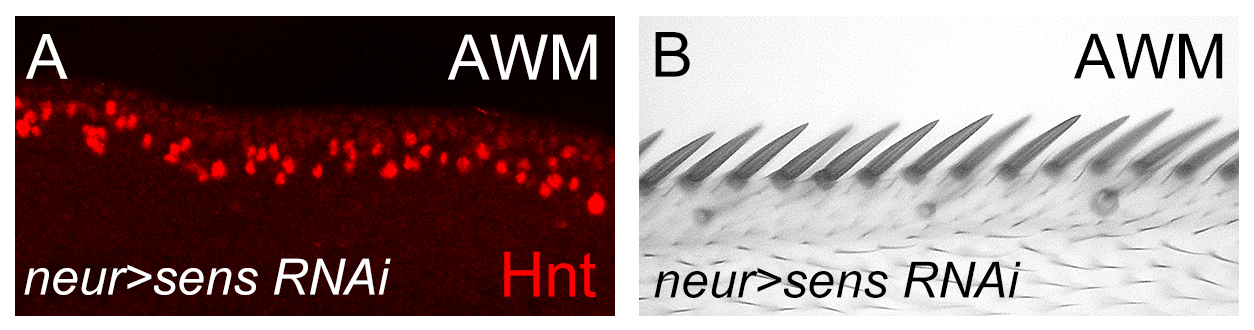

Supplement: Figure S7 — Reduction of Sens in bristle lineage cells does not affect innervated bristle formation at AWM. (A) High-level Hnt (red) was observed at AWM of sens knockdown wing disc 20–24 h APF by neur-Gal4. (B) Morphologically normal AWM bristles with sockets were observed at AWM of sens knockdown adult wing by neur-Gal4. (TIF) [file pgen.1004760.s007.tif]

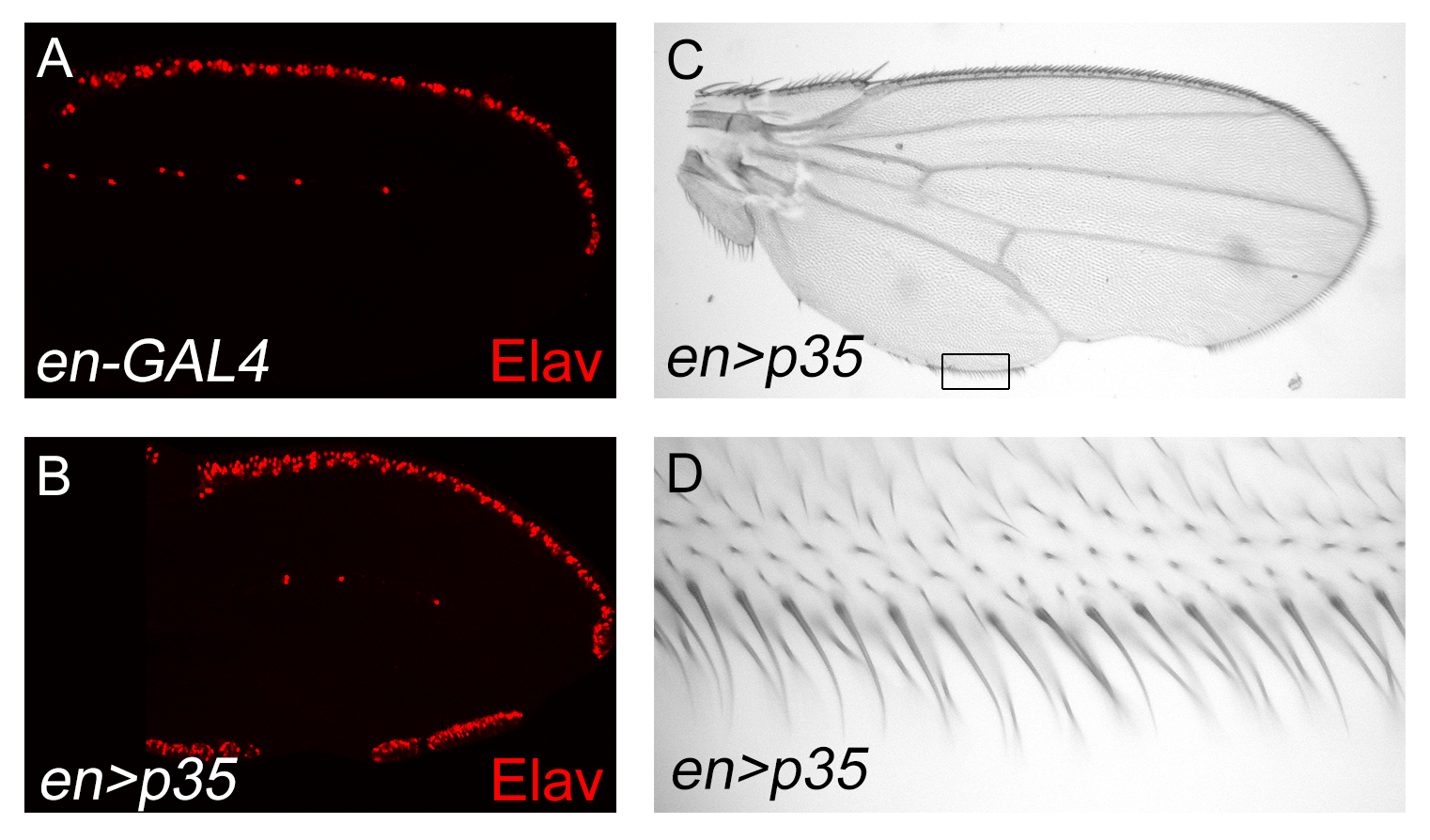

Supplement: Figure S8 — Inhibition of apoptosis does not affect the morphology of PWM bristles. (A) Neurons labeled by anti-Elav antibody (red) were detected at anterior but not posterior wing margin in control en-GAL4 wing disc 20–24 h APF. (B–D) Overexpression of anti-apoptotic protein p35 in the posterior compartment of the wing discs by en-GAL4. (B) Overexpression of p35 induced neurons (red) at PWM of 20–24 h APF wing disc. (C) Adult wing of p35 overexpression by en-GAL4. (D) Enlargement of the marked area in (C), showing formation of the morphologically normal PWM bristles with thin, long hair without socket support. (TIF) [file pgen.1004760.s008.tif]

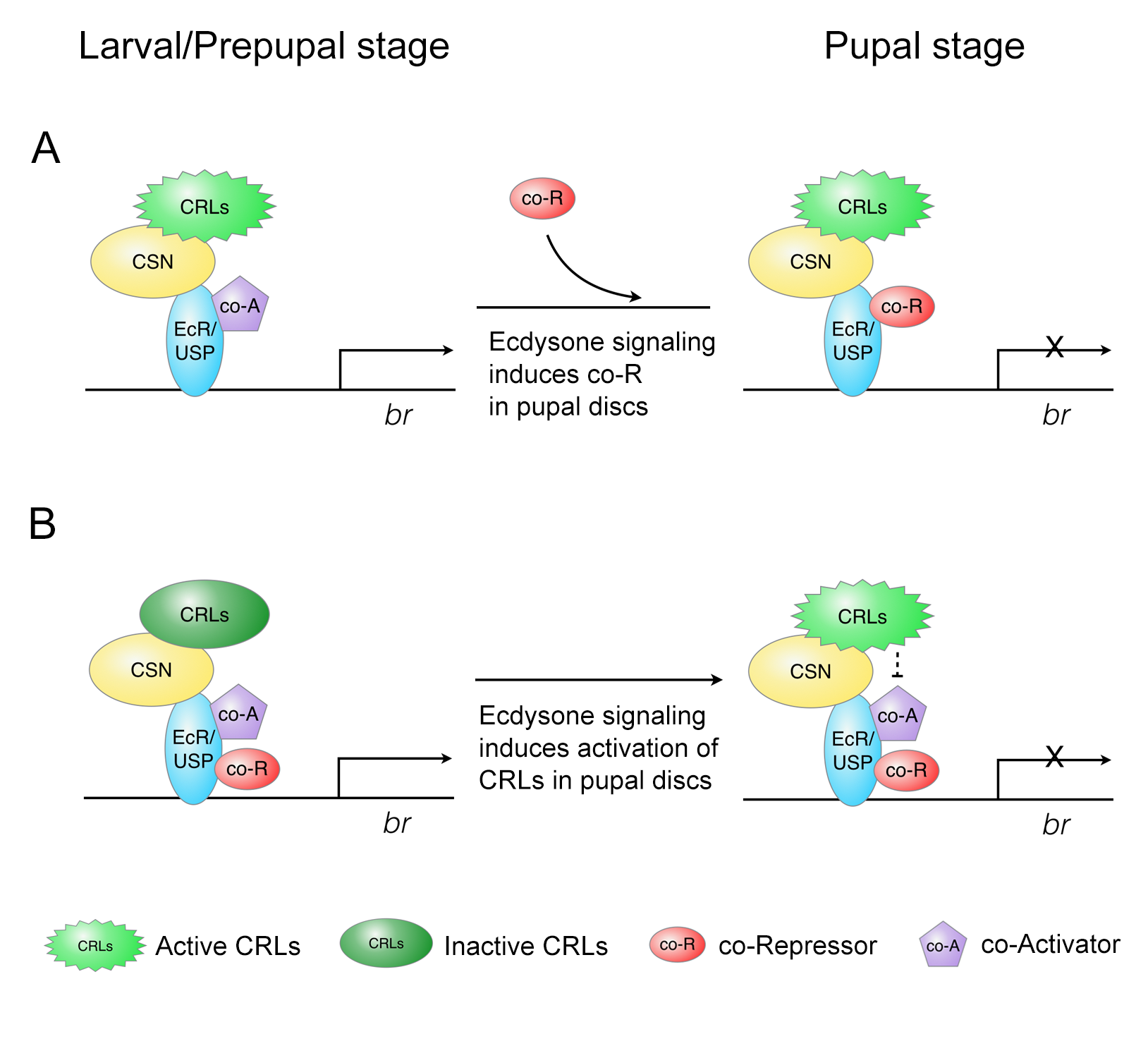

Supplement: Figure S9 — Proposed models for CSN-dependent switch of ecdysone receptor from activation to repression. We propose that the CSN, together with the cullin-based CRLs, is recruited to the br gene locus through association with EcR. While associated with EcR constitutively, the CSN-dependent pupa-specific switch of EcR to repressor may utilize two mechanisms: (A) the switch is mediated by induction of the specific co-repressor of EcR upon activation of ecdysone signaling at the prepupa-to-pupa transition. The co-repressor could replace the co-activator, a step facilitated by the nearby CRLs through a mechanism such as ubiquitination of co-activators for subsequent degradation. (B) The EcR switches from the activator to repressor through specific CRLs activated by ecdysone signaling at the prepupa-to-pupa transition. The CRL activation could be mediated through pupa-specific expression of substrate receptors or phosphorylation of the substrates to induce binding to the CRLs. These activated CRLs shut down br transcription through a mechanism such as inactivation of co-activators. Please see Discussion for more explanation of these models. (TIF) [file pgen.1004760.s009.tif]

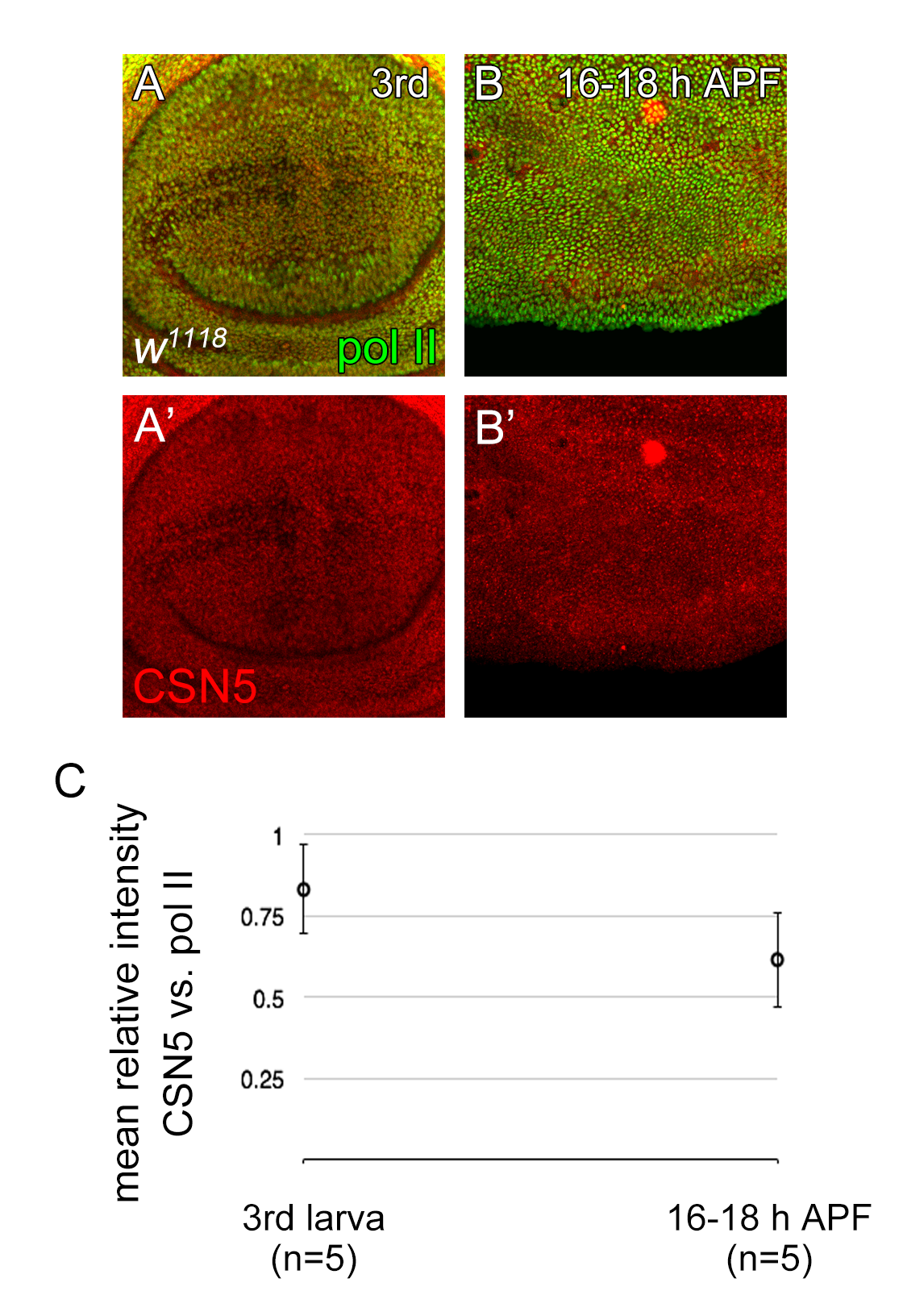

Supplement: Figure S10 — CSN5 is constitutively expressed in wing discs at late larval and pupal stages. (A–B′) CSN5 (red) detected by the anti-JAB1/CSN5 antibody was ubiquitously expressed in wild-type w1118 wing discs at late third instar larva (A, A′) and 16–18 h APF (B, B′). The co-stained anti-Pol II antibody staining (green) was used as a control for comparison of staining intensity in discs from different stages. (C) Diagram of the mean relative intensities of anti-CSN5 vs. co-stained anti-Pol II at late instar larva and 16–18 h APF. Five (N = 5) w1118 wing discs were scored at both time points. (TIF) [file pgen.1004760.s010.tif]

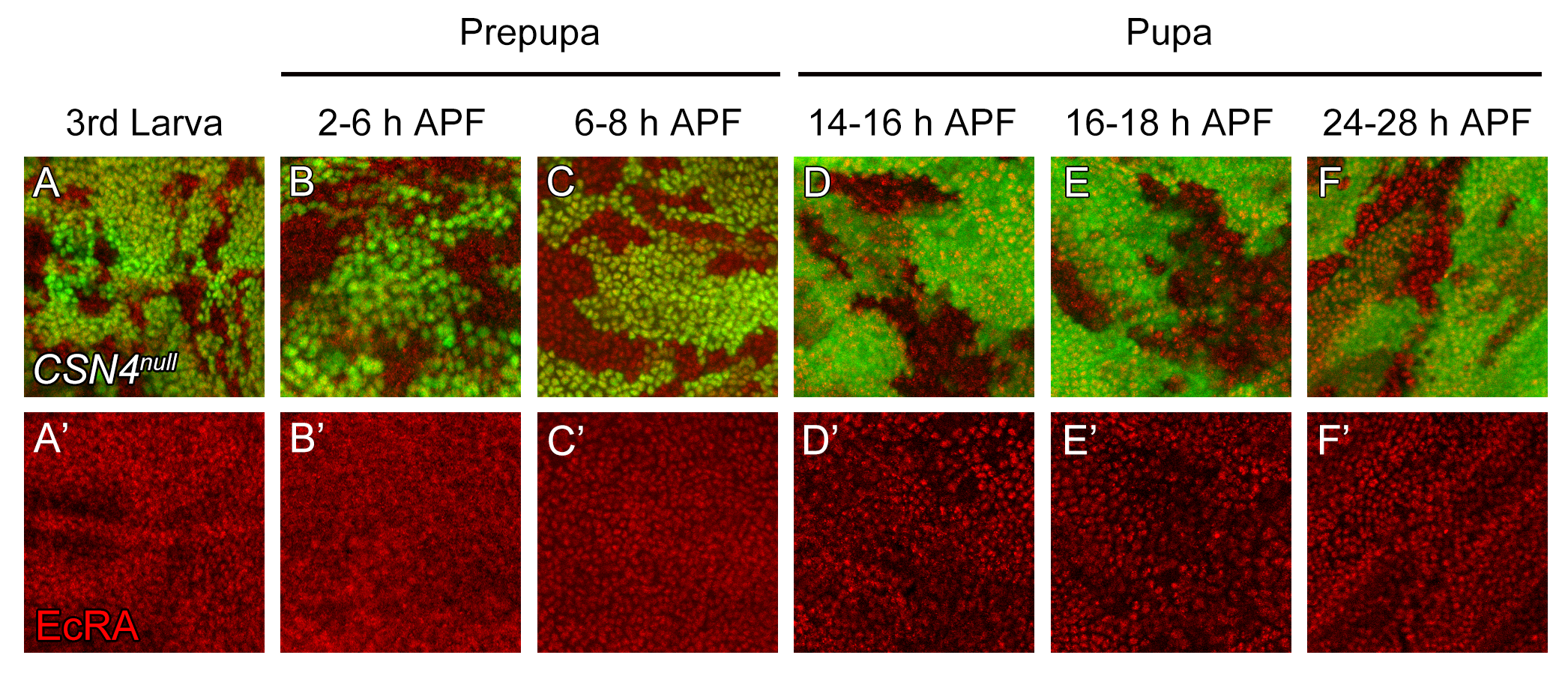

Supplement: Figure S11 — CSN does not regulate EcRA expression. (A–F′) EcRA (red) is constitutively expressed from late third instar larval to pupal stages, and its levels remained constant in CSN4null clones compared to the neighboring CSN4null/+ cells from late third larval instar to pupa. (TIF) [file pgen.1004760.s011.tif]
